# Selective Photocatalytic Reduction of Nitrobenzene to Aniline Using TiO_2_ Embedded in sPS Aerogel

**DOI:** 10.3390/polym15020359

**Published:** 2023-01-10

**Authors:** Wanda Navarra, Olga Sacco, Vincenzo Venditto, Vincenzo Vaiano

**Affiliations:** 1 Department of Chemistry and Biology “A. Zambelli”, INSTM Research Unit, University of Salerno, Via Giovanni Paolo II 132, 84084 Fisciano, Italy; 2 Department of Industrial Engineering, University of Salerno, Via Giovanni Paolo II 132, 84084 Fisciano, Italy

**Keywords:** TiO_2_, sPS aerogel, photoreduction, aniline, nitrobenzene

## Abstract

In recent years, aromatic substances have become the focus of environmental pollution-related concern due to their high stability and mutagenicity. In this regard, researchers have focused their attention on the development of photocatalytic processes to convert nitroaromatic compounds into aniline. In this work, the photocatalytic conversion of nitrobenzene (NB) to aniline (AN) was studied. The photocatalytic reaction was performed using commercial TiO_2_ (P25) and a photocatalytic aerogel, based on P25 embedded in syndiotactic polystyrene (sPS) aerogel (sPS/P25 aerogel) as photocatalysts. Different alcohols were used as hydrogen sources during the photocatalytic experiments. At the optimized operating conditions (photocatalysts dosage: 0.5 mg/L and 50% (*v*/*v*) EtOH%), an AN yield of over 99% was achieved. According to the results, this work could open avenues toward effective production of AN from NB using mild reaction conditions with sPS/P25 aerogel—in view of a possible scale-up of the photocatalytic process.

## 1. Introduction

Recently, contamination of the environment by organic contaminants has become a major global challenge. Among various organic contaminants and pollutants, nitroaromatic compounds caused concern [1,2], due to their toxicity and mutagenicity, as well as their high stability, low solubility, and intensive use as raw materials in industrial applications [3]. For this reason, different oxidation processes were employed for the complete degradation of these contaminants in nontoxic end products, such as carbon dioxide, nitrogen oxides, and water [4]. However, since these nitrated compounds are resistant to oxidative degradation, research has focused on possible alternatives to these environmental options. From this perspective, the conversion of nitroaromatic compounds into more added-value products could be a valid option. Indeed, aromatic amines, which are considered key intermediates in the synthesis of dyes, polymers, and many life-science products, including antioxidants, pharmaceuticals and agrochemicals [5,6,7,8], can be obtained by means of reduction reactions of nitroaromatic compounds.

Therefore, recent research has focused on finding suitable conversion processes for turning nitroaromatic compounds into amino derivatives, such as aniline (AN) [9]. On an industrial scale, AN is commonly synthesized by the catalytic hydrogenation of nitroaromatics, such as nitrobenzene (NB), in the liquid or vapor phase using high temperature, high H_2_ pressure, and the long reaction times required to achieve high selectivity for AN. Unfortunately, these synthesis processes are expensive and unsafe [10].

The most studied catalysts for the reduction of NB to AN are transition metals, such as Cu, and Ni [11,12], or noble metals, such as Pt, Pd, and Au [13]. Sn/HCl is employed commercially. However, there have been problems with waste disposal [14]. Therefore, the development of a sustainable catalytic process that could operate under mild reaction conditions by exploiting the mild reducing power of excited electrons was desirable [15]. Heterogeneous photocatalysis could be an attractive option, and has significant scientific value in ecological and green synthesis [16], as the reaction can be carried out using non-toxic metal oxides, such as TiO_2_, H_2_O, as solvents and alcohols as hydrogen source [17]. Indeed, when TiO_2_ is activated by light [18], the conduction band electrons reduce NB to AN [19] and, at the same time, the alcohol is oxidized to the corresponding aldehyde by the valence band positive holes [17]. Therefore, the use of H_2_ (as reducing agent) and toxic metals (as catalysts) could be avoided [14,15,16]. Literature studies showed that l TiO_2_ based photocatalysts were efficient in reducing NB to AN [20,21,22,23] under UV irradiation in slurry reactors, leading to the selective reduction of the only nitro group in the presence of suitable molecules acting as hole scavengers [22]. Although heterogeneous photocatalysis for this kind of reaction is very advantageous, one of the main drawbacks is the recovery of the powder catalyst from the reaction medium, which leads to an increase in the costs of the process—especially for a possible industrial scale-up of a photocatalytic system [24]. To overcome this limitation, the photocatalyst should be supported on materials with good chemical and mechanical stability, and be able to permanently immobilize the photocatalyst without decreasing the photocatalytic efficiency. Several papers reported that syndiotactic polystyrene-based polymer aerogels proved to be very interesting based on properties, such as their hydrophobicity, making them efficient as concentrators of organic molecules [25]. In recent years, syndiotactic polystyrene (sPS) aerogels, functionalized with TiO_2_ and ZnO-based photocatalysts, were effective in the degradation of water pollutants under UV and visible light irradiation [24,25,26]. However, to the best of our knowledge, these systems have yet to be studied for the photocatalytic reduction of NB to AN. For this reason this work presented a preliminary study of the photocatalytic conversion of NB to AN via photocatalysis, using an aerogel based on a commercial TiO_2_ powder photocatalyst (P25) embedded in sPS (sPS/P25).

## 2. Results and Discussion

### 2.1. Samples Characterization

**Figure 1:**
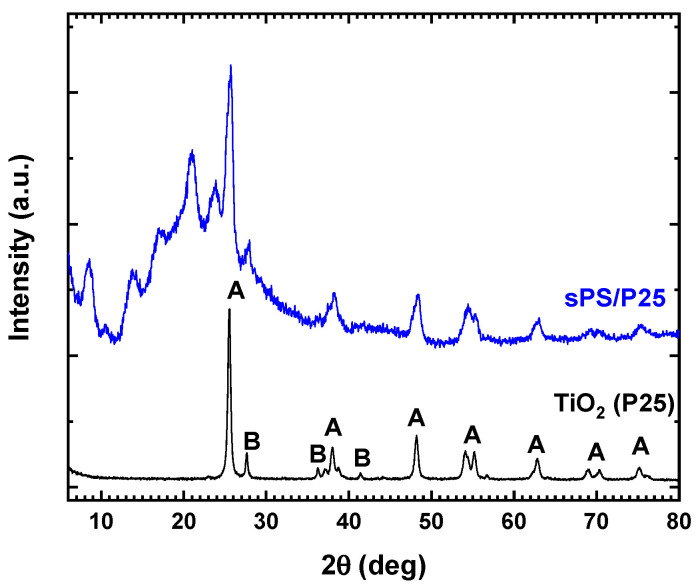
X-ray diffraction patterns of commercial TiO_2_ (P25) and sPS/P25 aerogel. A: anatase phase; B: rutile phase.

The X-ray diffraction patterns of TiO_2_ (P25) and sPS/P25 aerogel are reported in Figure 1. For TiO_2_ (P25), the typical reflexes of anatase and rutile crystalline phase were detected [27]. sPS/P25 aerogel showed peaks at 2θ = 8.3°, 13.7°, 16.7°, 20.7° and 23.6° due to the nanoporous crystalline phase of the sPS aerogel [24,25,26]. Additionally, the diffraction patterns assigned to both the anatase phase (at 25.30°, 37.17°, 37.93°, 38.68°, 47.05°, 54.05° 55.18° and 62.40°) and rutile phase (at 27.50°) of TiO_2_ (P25) were observed [28]. The results confirmed the successful incorporation of TiO_2_ (P25) particles inside the framework of sPS aerogel.

The specific surface area (SSA) values of TiO_2_ powder photocatalyst and sPS/P25 aerogel were also measured. For commercial TiO_2_, the SSA was about 50 m^2^/g, the typical value found for commercial P25 [29]. In contrast, the sPS/P25 aerogel had the typical aerogel values of sPS in δ-form [24,25,26,30], 250 m^2^/g.

### 2.2. Photocatalytic Activity Results on P25 in Powder Form

#### 2.2.1. Effect of the Reducing Agent

**Figure 2:**
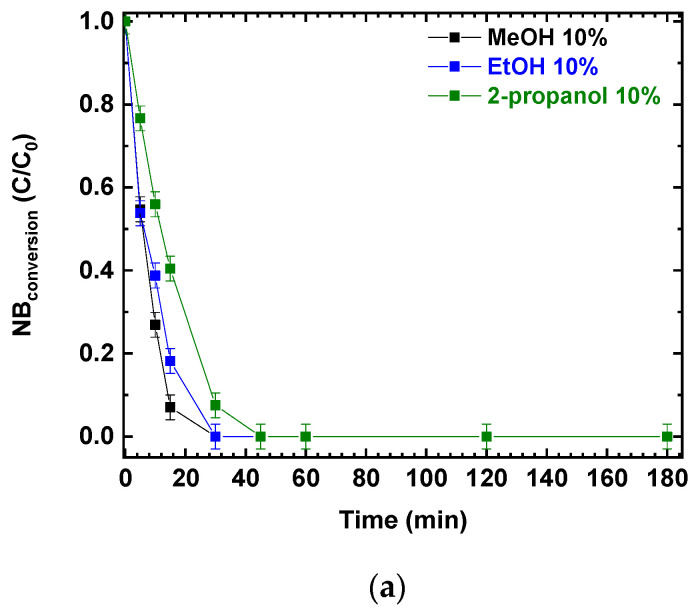

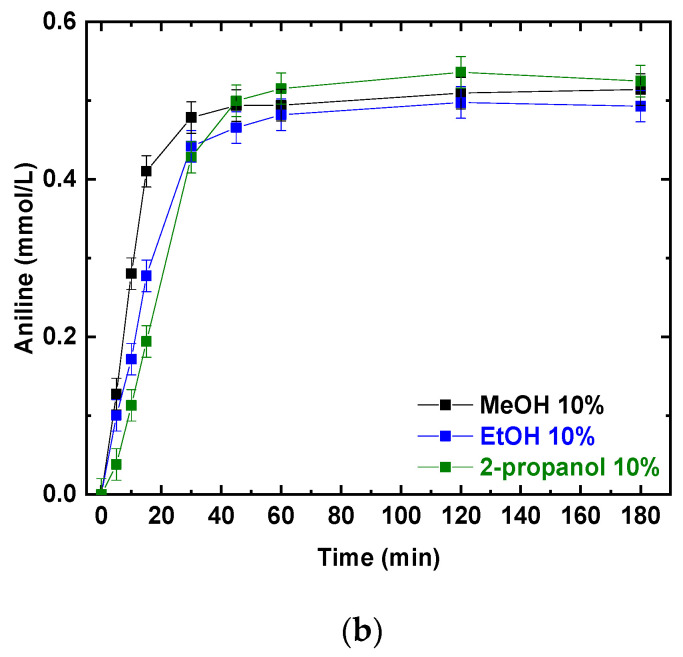
Comparison of the photocatalytic reduction of NB to AN using P25, with different reducing agents (MeOH, EtOH, 2-propanol): (**a**) NB conversion as a function of irradiation time; (**b**) AN production as a function of irradiation time. Reaction conditions: NB 1 mmol/L; water 100 mL; 0.5 g/L TiO_2_ (P25); temperature 25 °C; reaction time 180 min. Error bar ±0.02%.

Photocatalytic experiments were performed using P25 and different alcohols as hole scavengers. The photocatalytic NB reduction was tested using the same *v*/*v* percentage of methanol, ethanol, and 2-propanol; the results are presented in Figure 2a,b. NB conversions and AN production increased with the irradiation time. After 180 min, NB was completely converted (Figure 2a) and the final conversion was not influenced by the different reducing agents (MeOH, EtOH and 2-propanol). At the same time, aniline was produced, reaching a concentration of almost 0.5 mmol/L in all three scenarios (Figure 2b).

**Figure 3:**
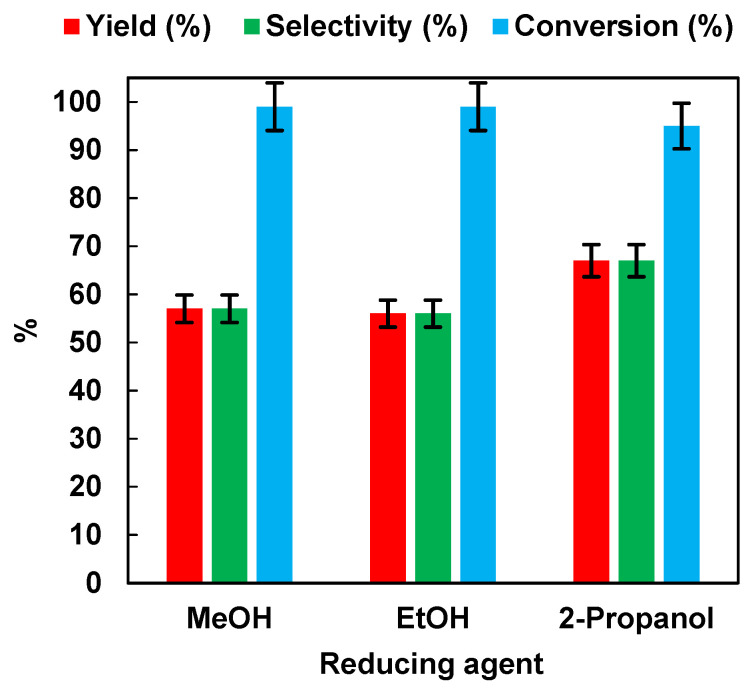
Effects of different hole scavengers on AN yield, AN selectivity, and NB conversion after 45 min of irradiation time. Error bar ±5%.

The results are summarized in Figure 3. It appeared that the reaction performance was not affected by the use of different alcohols in the aqueous solution. Indeed, after 45 min of irradiation time, the NB conversion values were 99%, 99%, and 95%, respectively, for MeOH, EtOH, and 2-propanol. AN yield and selectivity were 57% for MeOH and EtOH, and 67% for 2-propanol.

Although 2-propanol led to higher yield and selectivity values than MeOH and EtOH, the latter was preferred as a hydrogen source for environmental sustainability reasons. Indeed, environmental impact studies showed that using an H_2_O/Ethanol solution as a solvent blend in organic reactions was recommended over an H_2_O/2-propanol mixture [31].

#### 2.2.2. Effects of Initial EtOH Percentage

**Figure 4:**
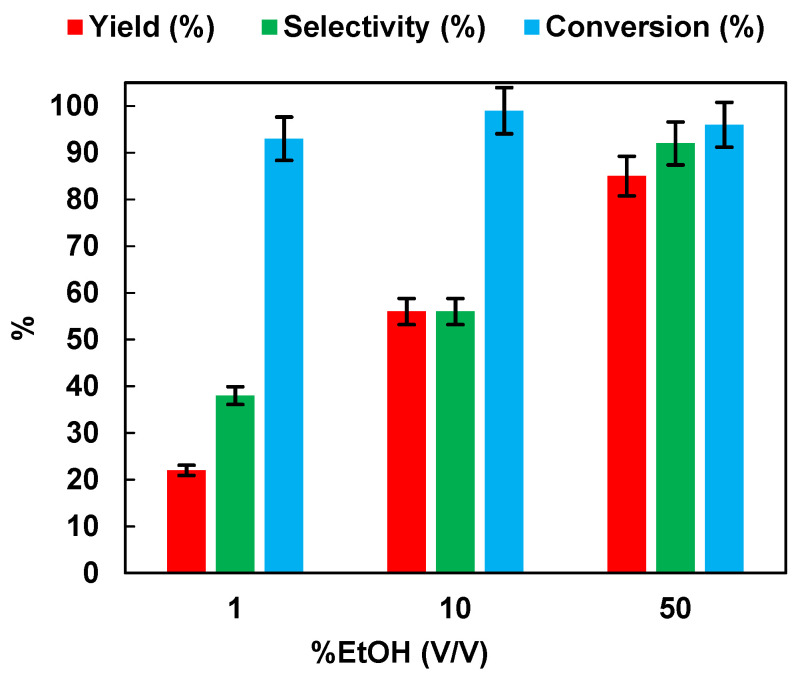
Effects of the EtOH% (*v*/*v*) in aqueous solution on the photocatalytic reduction of NB to AN using P25. Reaction conditions: NB 1 mmol/L; solution volume 100 mL; 0.5 g/L TiO_2_ (P25); temperature 25 °C; UV irradiation time t is 45 min. Error bar ±5%.

It has been documented in the literature that alcohols, in the photocatalytic conversion of NB, exert a double function; they act as a hydrogen source for the reduction and also as hole scavengers to inhibit the photoinduced electron hole-pairs [10,15,32]. Therefore, the effect of the initial ethanol percentage (*v*/*v*) on the photocatalytic reduction of NB to AN was assessed. The results are reported in Figure 4. Yield, selectivity, and conversion increased with the increase in EtOH percentage. At 50% of EtOH, 96% of NB was converted to AN, with a selectivity equal to 92%. 

**Figure 5:**
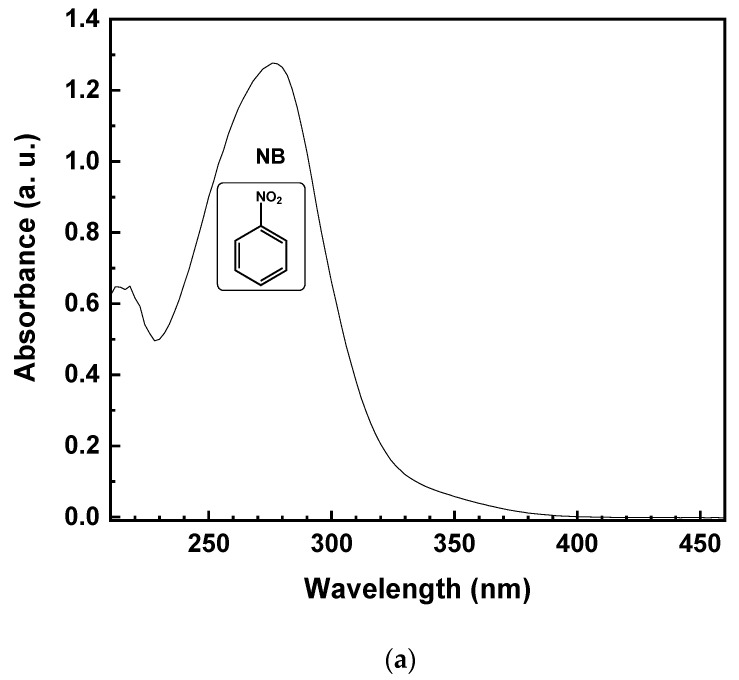

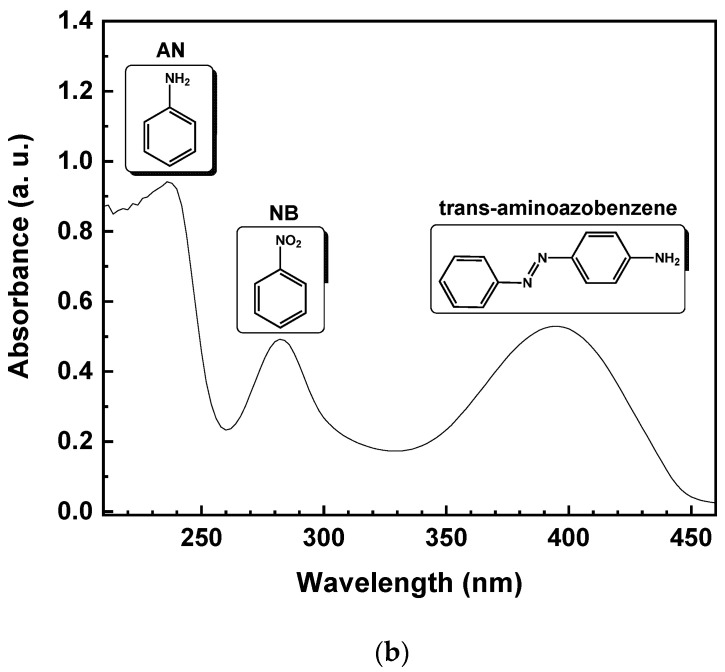
UV-vis spectrum of the aqueous solution with 10% EtOH (*v*/*v*) of NB initial solution (**a**) and after 15 min of UV irradiation time (**b**).

Theoretically, due to the lack of byproduct generation, the NB should be completely reduced to AN. Specifically, starting from 1 mmol/L of NB, the same amount of AN should be produced. Figure 5 reports the UV-vis spectrum of the reaction solution, with 10% of EtOH (*v*/*v*) after 15 min of irradiation time. The reduction of NB to AN was demonstrated by the absorbance peak, initially located at 277 nm (Figure 5a). After illumination with UV light, the formation of a band at 239 nm, corresponding to AN, was observed (Figure 5b). On the other hand, the lower selectivity toward AN, with the decreasing of EtOH percentage, was due to the appearance of a new band in the UV absorption spectrum at 395 nm (Figure 5b), corresponding to the formation of trans-4-aminoazobenzene (4-aminoAZ) [33].

**Figure 6:**
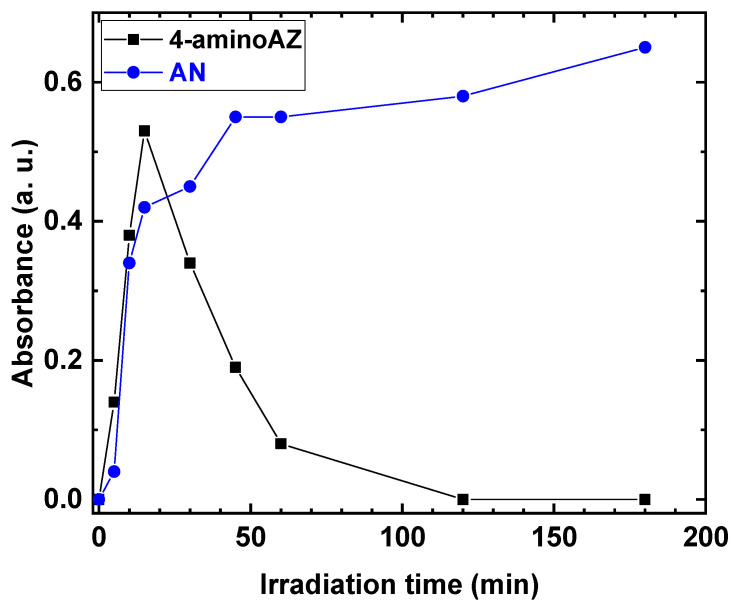
Absorbance of 4-aminoAZ and AN as a function of irradiation time.

The behavior of the 4-amino AZ and AN formation, as a function of the irradiation time, was investigated. The results are shown in Figure 6. 

The production of 4-amino AZ increased as the irradiation time increased, reaching a maximum after 15 min, and then gradually decreased during the reaction. In contrast, for AN, an increase in absorbance values was observed as the irradiation time increased, with a maximum absorbance of 0.6 at the end of the reaction. 

#### 2.2.3. Effect of Photocatalyst Dosage

**Figure 7:**
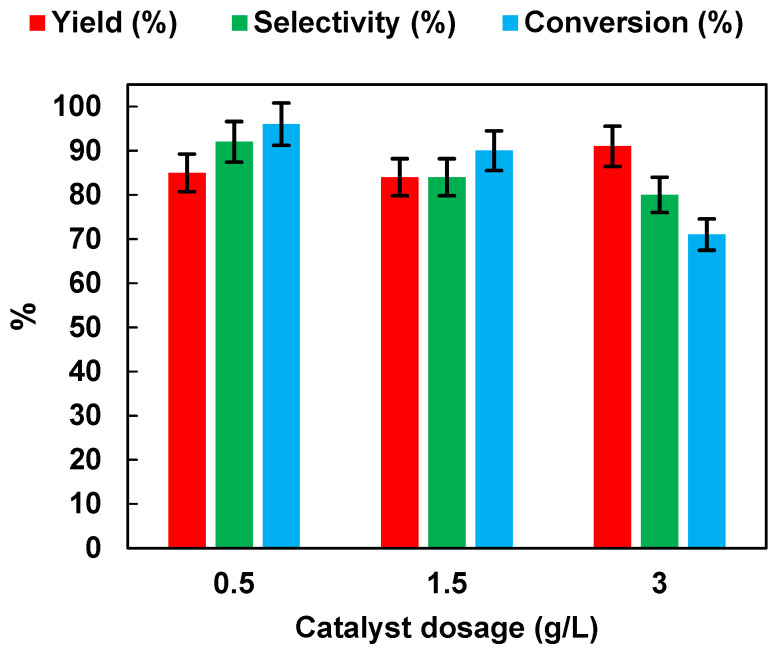
Effects of TiO_2_ (P25) dosage on AN yield, AN selectivity, and NB conversion. UV irradiation time t is 45 min. Error bar ±5%.

In order to choose the optimal photocatalyst concentration for NB reduction (initial NB concentration: 1 mmol/L), the effects of photocatalyst dosages were studied by performing experiments at different TiO_2_ (P25) dosages (in the range of 0.5–3 g/L) in the presence of EtOH (50% *v*/*v*). The results, in terms of yield, selectivity and conversion, are presented in Figure 7.

As the dosage of TiO_2_ (P25) increased from 0.5 g/L to 3 g/L, the NB yield remained unchanged, while selectivity to AN and NB conversion decreased from 92% to 80%, and from 96% to 71%, respectively. The results may have been related to the light-scattering and screening effects, due to the opacity of the suspension, which prevented proper illumination of the catalyst in solution [34]. The results may also have been related to the increase of aggregation phenomena causing a reduction in photocatalytic activity [35].

#### 2.2.4. Effect of Initial NB Concentration

**Figure 8:**
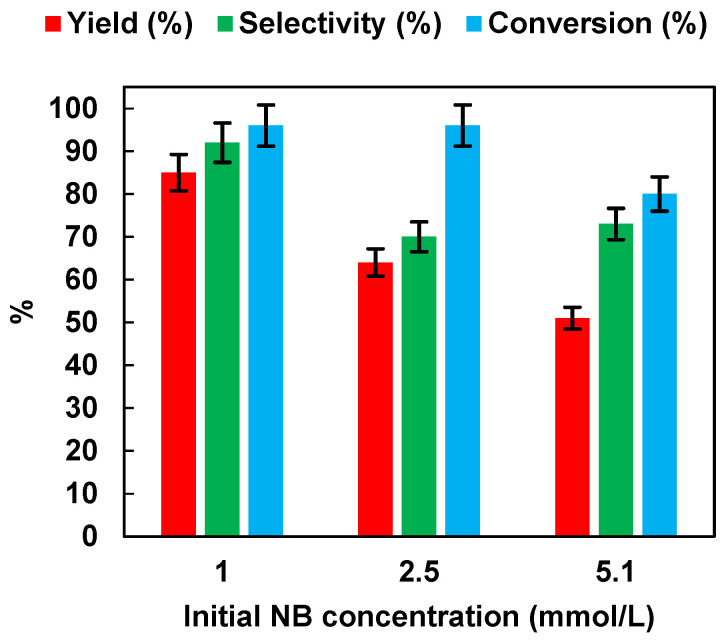
Effects of the initial NB concentration on AN yield, AN selectivity, and NB conversion. UV irradiation time t is 45 min. Error bar ±5%.

We investigated the effects of the initial NB concentration (1, 2.5 and 5.1 mmol/L) with the optimized catalyst dosage (0.5 g/L) on the photocatalytic performances. The results are reported in Figure 8. It was possible to observe that yield, selectivity, and conversion decreased with the increase of NB initial concentration from 1 up to 5.1 mmol/L. It is possible that the increase in concentration saturated the catalyst surface with NB molecules by preventing the adsorption of photons on the semiconductor surface to initiate photoreduction [36]. The above results proved that, in our study, for the reduction of the nitro group to the amino group using an NB concentration of 1 mmol/L, a photocatalyst dosage of 0.5 g/L and 50% EtOH as hydrogen source created suitable conditions for the reduction of nitro to the amino group using P25 as photocatalyst. Under these conditions, AN yield was higher than 99% after 3 h of irradiation. In Table 1, the photocatalytic NB reduction performances of different photocatalysts, as reported in the literature, are compared. It is worth noting that our optimized condition with P25 as catalyst showed a superior photocatalytic activity in comparison to other photocatalysts such as Pt-TiO_2_ [10], TiO_2_ [37], Ce_2_S_3_ [16], or even P25 itself [16], using a lower catalyst dosage and a lower irradiation time.

### 2.3. Photocatalytic Activity Results on sPS/P25 Aerogel

**Figure 9:**
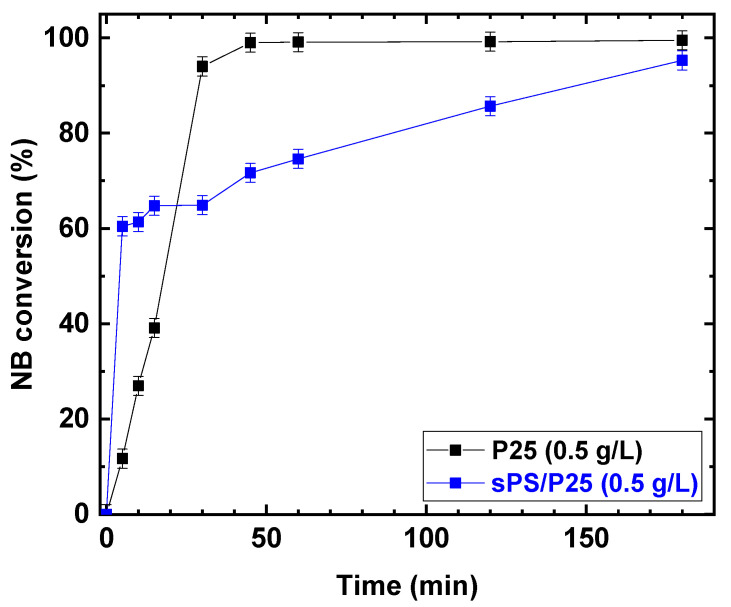
Comparison of the photocatalytic reduction of NB to AN with P25 and sPS/P25 aerogel. Reaction conditions: NB 1 mmol/L; water 100 mL; temperature 25 °C. Error bar ±2%.

Once the parameters of the photocatalytic reaction were optimized, P25 was embedded in the sPS aerogel and the photocatalytic reduction was carried out. The photocatalytic performances under UV irradiation for P25 and sPS/P25 aerogel are reported in Figure 9.

**Figure 10:**
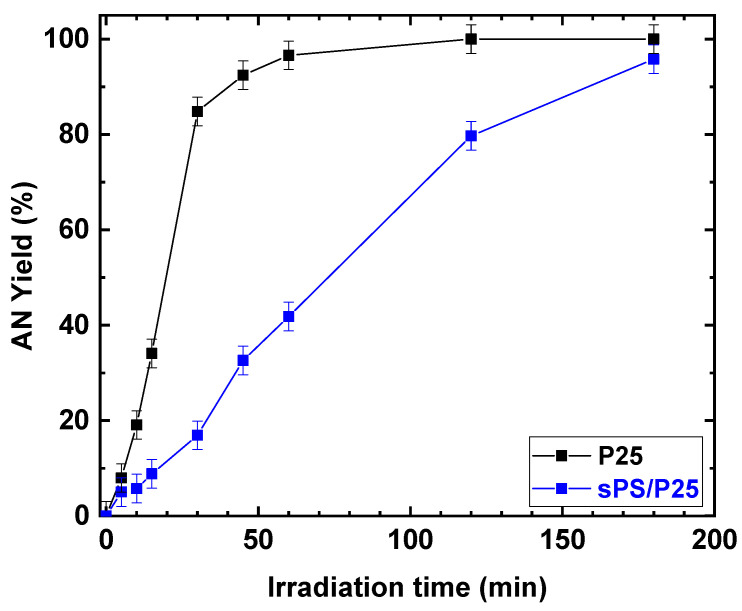
Comparison of the photocatalytic reduction of NB to AN with P25 and sPS/P25 aerogel. Reaction conditions: NB, 1 mmol/L; water, 100 mL; temperature, 25 °C. Error bar ±3%.

Despite the fact that the rates of increasing NB conversion for TiO_2_ (P25) powder and sPS/P25 aerogel were different, after 3 h of UV irradiation, similar conversion values were achieved for the P25 catalyst and sPS/P25 aerogel (99% and 95%, respectively). This resulted in the formation of AN as the only detectable reaction product with selectivity greater than 99% in both cases. In addition, the dependence of AN yield, as a function of irradiation time, was examined at an NB concentration of 1 mmol/L and EtOH percentage of 50% for P25 powder catalyst and sPS/P25 aerogel. The results are depicted in Figure 10. AN yield increased with reaction time within 180 min and, for both the tested samples, the AN yield was higher than 99%. 

## 3. Materials and Methods

### 3.1. Chemicals and Reagents

TiO_2_ (P25) particles were provided by Sigma-Aldrich. The syndiotactic polystyrene used for aerogel preparation was manufactured by Idemitsu Kosan Co., Ltd. (Chiyoda, Japan), under the trademark XAREC© 90ZC. The polymer was highly stereoregular with a content of syndiotactic triads over 98% (13C nuclear magnetic resonance data). Methanol (MeOH), Etanol (EtOH), and 2-propanol were purchased from Sigma-Aldrich, Milano, Italy.

### 3.2. Aerogel Preparation

A composite aerogel, based on sPS and P25 (sPS/P25 aerogel), was prepared according to the procedure reported by Sacco et al. [26]. In detail, syndiotactic polystyrene polymer and P25 photocatalyst with 95/5 weight ratio were dispersed in CHCl_3_ (solvent/sPS weight ratio in aerogel samples was 90/10), in a hermetically sealed test tube, and heated at 100 °C. The suspension was subsequently cooled to room temperature, forming a gel. The obtained gel was treated with supercritical carbon dioxide (using an ISCO SFX 220 extractor) for 4 h at T = 40 °C and P = 20 MPa to extract the solvent and obtain the relative monolithic composite aerogel. The sPS/P25 aerogel was a coherent phase in a cylindrical shape (diameter = 5.6 mm; height = 3 cm).

### 3.3. Samples Characterization

The BET method was used to evaluate the specific surface area by making dynamic N_2_ adsorption measurements at −196 °C using a Nova Quantachrome 4200e analyzer (Rivoli, Italy). X-ray diffraction (XRD) patterns were obtained with an automatic Bruker D2 Advance diffractometer, with reflection geometry and nickel-filtered Cu-Kα radiation. The intensities of XRD patterns were not corrected for polarization or Lorentz factors to allow easier comparison with most literature data. The acquisition interval ranged between 2θ = 5° and 90°, scanning with a step size of 0.0303° and an acquisition time of 0.200 s per point.

### 3.4. Photocatalytic Activity Tests

All the photocatalytic tests were performed using 100 mL of nitrobenzene (NB) aqueous solution. The reactor used for all the tests was a Pyrex cylindric photoreactor connected to a peristaltic pump (Watson-Marlow, Mazzano, Italy) to recirculate the solution. A UV-A LEDs strip, with emission at 365 nm and irradiance of 13 W/m^2^, was wrapped around the external surface of the reactor. During the photocatalytic experiment, N_2_ was bubbled inside the system, which was kept in the dark for 120 min to reach NB adsorption equilibrium on the catalyst surface and then irradiated for 180 min. At regular times, 2 mL of solution was collected and filtered. The products of the reaction were identified and quantified via gas chromatography-flame ionization detector (GC-FID) using an Agilent 7820A (Cernusco Sul Naviglio, Italy) equipped with a DB Heavy Wax capillary column (30 m × 320 µm × 0.25µm) under the following conditions: detector (FID) temperature = 300 °C; Oven = 60 °C hold 1 min; rate 25 up to 120 °C; equilibrate 3 min; rate 2 up to 210 °C; rate 25 up to 250 °C; injection volume 1 µL operated at 10:1 split mode. For the UV absorption spectrum of the intermediate (4-aminoAZ, Sigma-Aldrich, Milano, Italy), a fluorescence spectrometer, Duetta (Horiba Scientific, Torino, Italy), was used.

AN yield was calculated using the following formula:

Yield (%) = P_f_/C_i_ × 100 (1)

NB conversion was calculated by the following formula

Conversion (%) = (C_i_ − C_f_)/C_i_ × 100 (2)

where:

P_f_ is AN concentration measured at the generic irradiation time t;
C_i_ is the initial concentration of NB;
C_f_ is NB concentration measured at the generic irradiation time t.

The selectivity to AN in the reaction process was evaluated by the following formula:

Selectivity (%) = P_f_/(C_i_ − C_f_) × 100 (3)

## 4. Conclusions

A selective process for the photocatalytic reduction of NB under UV irradiation for AN production, in the presence of sacrificial electron donors, was developed. We investigated the effects of various experimental conditions—such as reducing agents, initial EtOH composition, photocatalyst dosage, initial NB concentration, and immobilization of the photocatalyst within sPS aerogel—on the reaction performances. Our study showed that aniline could be selectively obtained in the presence of P25 as photocatalysts, with yields greater than 99% with NB initial concentration of 1 mmol/L, photocatalyst dosage of 0.5 g/L, and 50% EtOH as the hydrogen source. Furthermore, it was shown that dispersing P25 within sPS aerogels after three hours of irradiation resulted in similar NB conversions of 99% and 95% for P25 and sPS/P25 aerogel, respectively, as well as AN production greater than 99%. The latter could be an optimal solution for a scale-up of the process since sPS/P25 aerogel can be easily removed from the solution, thereby avoiding a post-treatment step that would be necessary if a photocatalyst were used in powder form for the photoreaction.

## Figures and Tables

**Table 1:** Comparison with the available literature for photocatalytic conversion of NB.

Photocatalyst	Catalysts Dosage (g/L)	Reaction Time (h)	AN Yield (%)	Reducing Agent	Ref.
P25	0.5	3	>99	EtOH	Present work
P25	4	5	88	MeOH	[16]
P25	4	5	70	EtOH	[16]
P25	4	5	29	2-propanol	[16]
P25	3	3	~20	Glycerol	[10]
TiO_2_	4	6	89	MeOH	[37]
TiO_2_	4	6	71	EtOH	[37]
Pd-TiO_2_	3	3	~20	Glycerol	[10]
Ce_2_S_3_	4	5	44	MeOH	[16]
Ce_2_S_3_	4	5	38	EtOH	[16]
Ce_2_S_3_	4	5	20	2-propanol	[16]

## Data Availability

Not applicable here.
